# Physical manifestations of stress in women. Correlations between temporomandibular and pelvic floor disorders

**DOI:** 10.1371/journal.pone.0296652

**Published:** 2024-04-16

**Authors:** Isabel Mínguez-Esteban, Mónica De-la-Cueva-Reguera, Carlos Romero-Morales, Beatriz Martínez-Pascual, Jose A. Navia, María Bravo-Aguilar, Vanesa Abuín-Porras

**Affiliations:** 1 Faculty of Sport Sciences, Universidad Europea de Madrid, Villaviciosa de Odón, Madrid, Spain; 2 Musculoskeletal Pain and Motor Control Research Group, Faculty of Sport Sciences, Universidad Europea de Madrid, Madrid, Spain; 3 OnelifeCentre, Multidisciplinary Centre for the Prevention and Treatment of Pain, Alcorcón, Spain; 4 Facultad de Educación, Universidad de Alcalá, Alcalá de Henares, España; University of Rome, ITALY

## Abstract

**Introduction:**

Stress is characterized as a challenging occurrence that triggers a physiological and/or behavioral allostatic response, alongside the demands typically encountered throughout the natural course of life. A sustained state of stress gives rise to secondary effects, including insomnia and neck pain. Also, the risk of musculoskeletal problems in the cervical and lumbar spine can be increased due to a sustained state of stress. The present study main objective is to study the association between orofacial and pelvic floor muscles in women in Spain.

**Methodology:**

An observational, cross-sectional, retrospective analytical study was designed and carried out in the laboratories of the European University of Madrid. Sixty-five participants were recruited with a mean age of 29.9 ± 7.69. Measurements were taken by myotonometry on natural oscillation frequency (Hz), dynamic stiffness (N/m), elasticity (N/m2), mechanical stress relaxation time (ms) and creep, for the following muscles: right and left masseter, right and left temporalis and central fibrous nucleus of the perineum (CFPF). On the other hand, the subjects completed the following questionnaires: perceived stress scale (PSS-14), anxiety scale (STAI), self-reported bruxism questionnaire (CBA), Fonseca Anamnestic Index and the Pittsburgh Sleep Quality Scale (PSQI).

**Results:**

Significant correlations were found in several parameters between the right temporalis and CFPF (p<0.05). Highlighting the correlation between TMDs and lubrication r = -0.254 (p = 0.041) and bruxism with pain in sexual intercourse r = 0.261 (p = 0.036).

**Conclusion:**

The results support the proposed hypothesis. To the author’s knowledge, this is the first study which relates both locations, and it is suggested to continue with the research and expand the knowledge of it.

## 1. Introduction

Stress is defined as a threatening event that elicits a physiological and/or behavioral allostatic response in addition to those imposed by the normal life cycle [1] Stress can derive from extrinsic (people, environment, work) and intrinsic (personality, health) factors [2]. In 1986 when it was discovered that stress caused changes at the CNS level [3]. A sustained state of stress gives rise to secondary effects, including insomnia and neck pain among others [2]. In addition, sustained stress levels increase the risk of musculoskeletal problems in the cervical and lumbar spine [4]. Mental health is a global issue, it is estimated that the prevalence for stress is 36.5%, 26.9% for anxiety, 27.6% for sleep problems, 28.0% for depression and 50.0% for psychosocial distress [5].

Interest in the knowledge of the pelvic floor (PF) is currently booming. The assessment of the PF musculature, its tone and its properties to date are based on the subjective opinion of the clinician during palpation [6, 7]. Hypertonicity of the PF musculature is defined as an increased tone of the musculature in relation to contraction, rigidity, and its viscoelastic components, and may also be related to the pathologies previously mentioned [8, 9]. Dyspareunia, one of the consequences of hypertonic PF, is defined as pain during penetrative intercourse and affects approximately 14–34% of young women [10].

Temporomandibular disorders (TMDs) are composed of a group of pathologies that affect the orofacial region, such as bruxism [11]. They effect the temporomandibular joint, masticatory muscles and also related structures [12, 13]. TMDs negatively affect quality of life as they are considered one of the main causes of chronic orofacial pain [14]. In addition, psychological stress can alter masticatory muscle activity, which can lead to TMDs (15). The pain maintained over time can affect the central nervous system (CNS), thus explaining the distant pain associated with TMDs that many subjects suffer from [3, 15].

Advances in technology allow us to objectively assess muscle properties. Myotonometry is a reliable and non-invasive measurement method that objectively measures muscle properties by exerting a mechanical impulse on the muscle [16], calculating the following parameters: natural oscillation frequency (Hz), stiffness (N/m), elasticity (N/m2), relaxation time (ms) and creep [17, 18].

PF dysfunctions [19], TMDs [14] and stress, affect quality of life [2]. Likewise, all have a higher prevalence in the female sex [15, 20, 21]. In terms of age, those under 45 years of age present a higher degree of stress, and TMDs affect women of reproductive age to a greater degree [20].

Although there are studies that independently relate stress with the PF [21, 22] and stress with increased tone in the orofacial region [4, 23], no study has been found that proves whether there is a statistically significant relationship between the tone of both regions.

Therefore, the present study main objective is to study the association between orofacial and pelvic floor muscles in women in Spain. Consequently, the null hypothesis: There is no statistically significant relationship between external pelvic floor musculature, and orofacial musculature, stress, anxiety, bruxism, temporomandibular disorders, sleep quality and sexual dysfunction in healthy women aged 18–45 years in Spain.

## 2. Materials and methods

A retrospective, cross-sectional observational study was designed, following the Strobe criteria. Its objective was to study if there is an association between orofacial and pelvic floor musculature in women in Spain. The study was approved by the Ethics Committee from the Universidad Europea de Madrid (CPI/23.056), in January 2023. Obtained data have been processed following the 3/2018 law for Personal Data Protection and the declaration of Helsinki [24].

### 2.1 Participants

An online survey was designed to attract subjects via social media (facebook, Instagram…) and with printed material (flyers, posters) distributed in targeted areas where potential participants could receive the information. A total of 95 subjects answered the online survey from February 20^th^ to June 1st 2023. Written informed consent was required from participants prior to data collection. After applying exclusion criteria, the final number of participants was 65. Inclusion criteria were: a) women aged 18–45 years, b) residents in Spain. And the exclusion criteria: a) pregnant women, b) suffering from diseases that alter the tone of the musculature such as: diseases of the nervous system, c) muscular dystrophy in the orofacial region and PF, d) neurodegenerative diseases and muscular diseases (fibromyalgia, ruptures in the musculature to be assessed), e) bruxism diagnosed, f) having suffered a serious trauma in the last 6 months, g) PF pathologies: stress urinary incontinence, overflow, urgency and prolapse, as well as having active urinary infections such as chlamydia, herpes, HIV, h) use of braces or Invisalign.

### 2.2 Outcome measures

The measurements were conducted at the University European of Madrid.

The measurement was divided into two parts. First, the MyotonPro [16] was used for the left and right masseters, left and right temporalis and the CFPF. During the measurements of the masseters and temporalis the basin should be placed in the supine position. The location of measurements for the masseters; two finger-widths above the arch of the jaw [25]. In the case of the temporalis, the measurement site was taken as a reference according to electromyography measurements, described by the SENIAM program [26]. For CFPF, the long probe, nitrile gloves and the probe cover were used. The patient, in supine decubitus, wearing his clothes. The CFPF was located, and the probe was placed [27].

Once measurements were concluded, the participants received a questionnaire which included the following validated questionnaires: Perceived stress questionnaire (PSS-14) [28], Self-Reported Bruxism Questionnaire [29], Female Sexual Function Index (FSFI) [30, 31], Pittsburgh Sleep Quality Index [32, 33] (PSQI), STAI [34, 35] Fonseca Anamnestic Index [36].

### 2.3 Statistical analysis

The Jamovi V.2.3 database and SPSS V.20 were used for data analysis. Normality, correlation, heterogeneity and collinearity were controlled. The descriptive variable, age, was studied by descriptive analysis and the mean and standard deviation were found. In relation to the proposed quantitative variables, it was considered by the central limit theorem [37] that the present study (n = 65) follows a normal distribution. Pearson’s correlation matrix was used to study the correlation between the different variables. A priori analysis with G*Power with [38] α = 0.05, β = 0.2 and effect size |ρ| = 0.3 recommended a sample size of 64 participants for correlations.

## 3. Results

The results obtained after performing the statistical analysis to test the hypotheses are as follows.

The descriptive analysis of the variable age was carried out in the 65 study subjects, with a mean age of 29.9 ± 7.69.

Pearson correlation analysis was performed between left temporal and CFPF with respect to the same variables described above, and no statistically significant correlations were found (p>0.05) (Tables 1–3).

**Table 1:** Correlation matrix left masseter muscle–Central fibrous nucleus of the perineum (CFNP).

		LMMF (Hz)	LMMS (N/m)	LMME (N/m^2^)	LMMR (ms)	LMMC	CFNPf (Hz)	CFNPs (N/m)	CFNPe (N/m^2^)	CFNPr (ms)	CFNPc
**CFNPf (Hz)**	Pearson Correlation	0.031	0.112	-0.130	-0.061	-0.068	—				
	P value	0.808	0.376	0.301	0.629	0.590	—				
**CFNPs (N/m)**	Pearson Correlation	-0.007	0.054	-0.068	-0.025	-0.034	**0.924** ***	—			
	P value	0.958	0.668	0.593	0.846	0.789	< .001	—			
**CFNPe (N/m^2^)**	Pearson Correlation	0.025	0.094	-0.054	-0.024	-0.021	**0.628** ***	0.541***	—		
	P value	0.844	0.456	0.670	0.851	0.865	< .001	< .001	—		
**CFNPr (ms)**	PearsonCorrelation	-0.014	-0.087	0.059	0.051	0.057	**-0.895** ***	**-0.890** ***	**-0.583** ***	—	
	P value	0.914	0.489	0.640	0.684	0.651	< .001	< .001	< .001	—	
**CFNPc**	Pearson Correlation	0.138	0.170	0.069	-0.122	-0.119	**-0.554** ***	**-0.547** ***	-0.338**	**0.767** ***	—
	P value	0.271	0.175	0.585	0.332	0.345	< .001	< .001	0.006	< .001	—

LMMF: left masseter muscle frequency, LMMS: left masseter muscle stiffness, LMME: left masseter muscle elasticity, LMMR: left masseter muscle relaxation, LMMC: left masseter muscle creep. CFNPf: central fibrous nucleus of the perineum frequency, CFNPs: central fibrous nucleus of the perineum stiffness, CFNPe: central fibrous nucleus of the perineum elasticity, CFNPr: central fibrous nucleus of the perineum relaxation, CFNPc: central fibrous nucleus of the perineum creep.

Pearson Correlation

* p < .05

** p < .01

*** p < .001

**Table 2:** Correlation matrix right masseter muscle–Central fibrous nucleus of the perineum (CFNP).

		RMMF(Hz)	RMMS (N/m)	RMME (N/m^2^)	RMMR (ms)	RMMC	CFNPf (Hz)	CFNPs (N/m)	CFNPe (N/m^2^)	CFNPr (ms)	CFNPc
**CFNPf (Hz)**	Pearson Correlation	0.051	-0.007	-0.136	-0.081	-0.078	—				
	P value	0.685	0.953	0.281	0.519	0.538	—				
**CFNPs (N/m)**	Pearson Correlation	0.020	-0.054	-0.163	-0.008	-0.009	**0.924** ***	—			
	P value	0.876	0.670	0.193	0.951	0.941	< .001	—			
**CFNPe (N/m^2^)**	Pearson Correlation	0.126	0.058	-0.097	-0.112	-0.048	**0.628** ***	**0.541** ***	—		
	P value	0.317	0.647	0.442	0.374	0.707	< .001	< .001	—		
**CFNPr (ms)**	Pearson Correlation	0.004	0.061	0.118	0.017	-0.002	**-0.895** ***	**-0.890** ***	**-0.583** ***	—	
	P value	0.972	0.627	0.348	0.892	0.990	< .001	< .001	< .001	—	
**CFNPc**	Pearson Correlation	0.128	0.172	0.080	-0.109	-0.129	**-0.554** ***	**-0.547** ***	**-0.338** ***	**0.767** ***	—
	P value	0.310	0.170	0.527	0.387	0.307	< .001	< .001	0.006	< .001	—

RMMF: right masseter muscle frequency, RMMS: right masseter muscle stiffness, RMME: right masseter muscle elasticity, RMMR: right masseter muscle relaxation, RMMC: right masseter muscle creep. CFNPf: central fibrous nucleus of the perineum frequency, CFNPs: central fibrous nucleus of the perineum stiffness, CFNPe: central fibrous nucleus of the perineum elasticity, CFNPr: central fibrous nucleus of the perineum relaxation, CFNPc: central fibrous nucleus of the perineum creep.

Pearson Correlation

* p < .05

** p < .01

*** p < .001

**Table 3:** Correlation matrix left temporalis muscle–Central fibrous nucleus of the perineum (CFNP).

		MTTL(Hz)	MTSL (N/m)	MTEL (N/m^2^)	MTRL (ms)	MTCL	CFNPf (Hz)	CFNPs (N/m)	CFNPe (N/m^2^)	CFNPr (ms)	CFNPc
**CFNPf (Hz)**	Pearson Correlation	0.051	-0.007	-0.136	-0.081	-0.078	—				
	P value	0.685	0.953	0.281	0.519	0.538	—				
**CFNPs (N/m)**	Pearson Correlation	0.020	-0.054	-0.163	-0.008	-0.009	**0.924** ***	—			
	P value	0.876	0.670	0.193	0.951	0.941	< .001	—			
**CFNPe (N/m^2^)**	Pearson Correlation	0.126	0.058	-0.097	-0.112	-0.048	**0.628** ***	**0.541** ***	—		
	P value	0.317	0.647	0.442	0.374	0.707	< .001	< .001	—		
**CFNPr (ms)**	PearsonCorrelation	0.004	0.061	0.118	0.017	-0.002	**-0.895** ***	**-0.890** ***	**-0.583** ***	—	
	P value	0.972	0.627	0.348	0.892	0.990	< .001	< .001	< .001	—	
**CFNPc**	Pearson Correlation	0.128	0.172	0.080	-0.109	-0.129	**-0.554** ***	**-0.547** ***	**-0.338** ***	**0.767** ***	—
	P value	0.310	0.170	0.527	0.387	0.307	< .001	< .001	0.006	< .001	—

LTMF: left temporalis muscle frequency, LTMS: left temporalis muscle stiffness, LTME: left temporalis muscle elasticity, LTMR: left temporalis muscle relaxation, LTMC: left temporalis muscle creep. CFNPf: central fibrous nucleus of the perineum frequency, CFNPs: central fibrous nucleus of the perineum stiffness, CFNPe: central fibrous nucleus of the perineum elasticity, CFNPr: central fibrous nucleus of the perineum relaxation, CFNPc: central fibrous nucleus of the perineum creep.

Pearson Correlation

* p < .05

** p < .01

*** p < .001

Regarding the relationship between right temporal and CFPF, it has been observed that there are significant correlations in: right temporal relaxation time and CFPF stiffness being its value of r = 0.253 (p = 0.042); right temporal relaxation time and CFPF relaxation time r = -0. 261 (p = 0.036); right temporal creep and CFPF stiffness r = 0.262 (p = 0.035); right temporal creep and CFPF elasticity r = 0.282 (p = 0.023), resulting the rest of the correlated variables not statistically significant p>0.05 (Table 4).

**Table 4:** Correlation matrix right temporalis muscle–Central fibrous nucleus of the perineum (CFNP).

		MTTR(Hz)	MTSR (N/m)	MTER (N/m^2^)	MTRR (ms)	MTCR	CFNPf (Hz)	CFNPs (N/m)	CFNPe (N/m^2^)	CFNPr (ms)	CFNPc
**CFNPf (Hz)**	Pearson Correlation	0.051	-0.007	-0.136	-0.081	-0.078	—				
	P value	0.685	0.953	0.281	0.519	0.538	—				
**CFNPs (N/m)**	Pearson Correlation	0.020	-0.054	-0.163	-0.008	-0.009	**0.924** ***	—			
	P value	0.876	0.670	0.193	0.951	0.941	< .001	—			
**CFNPe (N/m^2^)**	Pearson Correlation	0.126	0.058	-0.097	-0.112	-0.048	**0.628** ***	**0.541** ***	—		
	P value	0.317	0.647	0.442	0.374	0.707	< .001	< .001	—		
**CFNPr (ms)**	PearsonCorrelation	0.004	0.061	0.118	0.017	-0.002	**-0.895** ***	**-0.890** ***	**-0.583** ***	—	
	P value	0.972	0.627	0.348	0.892	0.990	< .001	< .001	< .001	—	
**CFNPc**	Pearson Correlation	0.128	0.172	0.080	-0.109	-0.129	**-0.554** ***	**-0.547** ***	**-0.338** ***	**0.767** ***	—
	P value	0.310	0.170	0.527	0.387	0.307	< .001	< .001	0.006	< .001	—

RTMF: right temporalis muscle frequency, RTMS: right temporalis muscle stiffness, RTME: right temporalis muscle elasticity, RTMR: right temporalis muscle relaxation, RTMC: right temporalis muscle creep. CFNPf: central fibrous nucleus of the perineum frequency, CFNPs: central fibrous nucleus of the perineum stiffness, CFNPe: central fibrous nucleus of the perineum elasticity, CFNPr: central fibrous nucleus of the perineum relaxation, CFNPc: central fibrous nucleus of the perineum creep.

Pearson Correlation

* p < .05

** p < .01

*** p < .001

The following variables were correlated: frequency, rigidity, elasticity, relaxation time and creep of the CFPF in relation to the following questionnaires: stress (PSS-14), anxiety (STAI), bruxism (CBA), TMDs (Fonseca Amnesic Index) and sleep quality (Pittsburg Sleep Quality Index Scale), and no statistically significant correlations were found (p>0.05). It should be noted that the correlation value obtained between CFPF rigidity and bruxism was close to the significance value (Table 5). Statistically significant relationships were found between the questionnaires mentioned above (p<0.05) (Table 6).

**Table 5:** Correlation of the perineum (CFNP) and questionaries: Stress, anxiety, bruxism, TMD, sleep quality.

		CFNPf (Hz)	CFNPs(N/m)	CFNPe (N/m^2^)	CFNPr (ms)	CFNPc	Stress (PSS-14)	Anxiety (STAI)	Bruxism (CBA)	TMD (Fonseca Index))	Sleep Quality (Pittisburg)
**CFNPf (Hz)**	Pearson Correlation	1	,924***	**,628** **	**-,895** **	**-,554** **	-,005	,154	-,051	,074	-0,75
	P value		,000	,000	,000	,000	,968	,221	,221	,558	,550
**CFNPs (N/m)**	Pearson Correlation	**,924** ***	1	**,541** **	-,890	**-,547** **	,060	,242	-,092	,050	,025
	P value	,000		,000	,000	,000	,637	,052	,467	,693	,842
**CFNPe (N/m^2^)**	Pearson Correlation	**,628** **	,541**	1	**-,583** **	**-,338** **	,080	,026	-,037	-,004	-,157
	P value	,000	,000		,000	,006	,535	,835	,771	,977	,842
**CFNPr (ms)**	Pearson Correlation	**-,895** **	**-,890** **	-,583**	1	**,767** **	-,010	-,130	,050	-,0122	-,045
	P value	,000	,000	,000		,000	,939	,302	,690	,331	,723
**CFNPc**	Pearson Correlation	**-,554** **	**-,547** **	**-,338** **	**,767** **	1	,047	-,027	,143	-,104	-,074
	P value	,000	,000	,000	,000		,713	,830	,254	,408	,559
**Stress (PSS-14)**	Pearson Correlation	-,005	,060	,080	-,010	,047	1	**,736** **	,235	,255*	**,274** *
	P value	,968	,637	,525	,939	,713		,000	,059	,040	,027
**Anxiety (STAI)**	Pearson Correlation	,154	,242	,026	-,130	-,027	**,736** **	1	,245*	**,273** *	,240
	P value	,221	,052	,835	,302	,830	,000		,050	,028	,054
**Bruxism (CBA)**	Pearson Correlation	-,0,51	-,092	-,037	,050	,143	,235	**,245** *	1	**,752** **	-0,19
	P value	,685	,467	,771	,690	,254	,059	,050		,000	,881
**TMD (Fonseca Index))**	Pearson Correlation	,074	,050	-,004	-,122	-,104	,255*	,255*	**,752** **	1	**,250** *
	P value	,558	,693	,977	,331	,408	,040	,040	,000		,044
**Sleep Quality (Pittisburg)**	Pearson Correlation	-0,75	,025	-,157	-0,45	-0,74	**,274** *	**,274** *	-,019	**,250** *	1
	P value	,550	,842	,212	,723	,559	,027	,027	,881	,044	

Correlation matrix between PPS-14 (Perceived Stress Scale), STAI (State-Trait Anxiety Index), Bruxism, CBA questionnaire, Fonseca’s Anamnesis Index (TMD: temporomandibular disorders), Pittishburg Sleep Quality Index and FSFI (Female Sexual Function Index) and CFNPf: central fibrous nucleus of the perineum frequency, CFNPs: central fibrous nucleus of the perineum stiffness, CFNPe: central fibrous nucleus of the perineum elasticity, CFNPr: central fibrous nucleus of the perineum relaxation, CFNPc: central fibrous nucleus of the perineum creep.

Pearson Correlation

* p < .05

** p < .01

*** p < .001

**Table 6:** Correlation central fibrous nucleus of the perineum (CFNP) and FSIF questionnaire and its domains.

		CFNPt	CFNPs	CFNPe	CFNPr	CFNPc	Desire	Lubrication	Orgasm	Satisfaction	Pain	FSIF
**CFNPt**	Pearson Correlation	1	**,924** **	**-,628** **	**-,895** **	**-,554** **	,028	-,043	-,011	-,169	-,137	-,124
	P value		,000	,000	,000	,000	,826	,732	,931	,187	,276	,385
**CFNPs**	Pearson Correlation	,924**	1	,541**	-,890	**,547** **	,047	-,088	-,063	-,220	-,174	-,161
	P value	,000		,000	,000	,000	,710	,488	,616	,083	,165	,259
**CFNPe**	Pearson Correlation	,628**	,541	1	**-,583** **	**-,338** **	,019	-,065	,040	-0,17	-,118	,058
	P value	,000	,000		,000	,006	,878	,609	,753	,894	,350	,687
**CFNPr**	Pearson Correlation	**-,895** **	-,890	**-,583** **	1	**,767** **	-,169	-,023	-,054	,046	,096	-,032
	P value	,000	,000	,000		,000	,179	,854	,671	,723	,447	,825
**CFNPc**	Pearson Correlation	**-,554** **	-,547	**-,338** **	,767**	1	-,240	-,154	-,171	-,145	-,042	-,128
	P value	,000	,000	,000	,000		,054	,220	,174	,257	,738	,370
**Desire**	Pearson Correlation	,028	,047	,019	-,169	-,240	1	,499**	,614**	,552**	,069	,788**
	P value	,826	,710	,878	,179	,054		,000	,000	,000	,585	,000
**Lubrication**	Pearson Correlation	-,043	-,088	-,065	-,023	-,154	,499**	1	,886**	,793**	,370**	**,585** **
	P value	,732	,488	,609	,854	,220	,000		,000	,000	,000	,000
**Orgasm**	Pearson Correlation	-,011	-,063	,040	-,054	-,171	,614**	**,886** **	1	,839**	,313*	,813**
	P value	,931	,488	,753	,671	,174	,000	,000		,000	,011	,000
**Satisfaction**	Pearson Correlation	-,196	-,220	-,017	,046	-,145	,552**	**,793** **	,839**	1	,454**	,808**
	P value	,187	,083	,894	,723	,257	,000	,000	,000		,000	,000
**Pain**	Pearson Correlation	-,137	-,174	-,118	,096	-,042	,069	,370**	,313**	**,454** **	1	-,141
	P value	,276	,165	,350	,447	,738	,585	,000	,000	,000		,324
**FSIF**	Pearson Correlation	-,124	-,161	,058	-,032	-,128	,788**	,585**	,813**	,808**	-,141	1
	P value	,385	,259	,687	,825	,370	,000	,000	,000	,000	,324	

Correlation between CFNPt: central fibrous nucleus of the perineum tone, CFNPs: central fibrous nucleus of the perineum stiffness, CFNPe: central fibrous nucleus of the perineum elasticity, CFNPr: central fibrous nucleus of the perineum relaxation, CFNPc: central fibrous nucleus of the perineum creep and FSFI (Female Sexual Function Index) and its domains: desire, lubrication, orgasm, satisfaction and pain

Pearson Correlation

* p < .05

** p < .01

*** p < .001

Out of 65 participants, 17 subjects did not have sexual intercourse, considering the sample of n = 48 for the statistical study of the sample. The CFPF and the IFSF domains (desire, arousal, lubrication, orgasm, satisfaction, and pain) were correlated. Similar, the relationship between the domains was studied (Table 6).

Finally, the relationship between the questionnaires of stress, anxiety, bruxism, TMDs and sleep quality with the sexual function domains of the IFSF was tested. We can highlight that sleep quality is related to arousal, lubrication, and orgasm at any significance level 0.05 (0.01). The estimate of these correlations are r = -0.352 (p = 0.006), r = - 0.348 (p = 0.004) and r = -0.310 (p = 0.012) respectively. To interpret these correlations, it is important to keep in mind that, in terms of sleep quality, the lower the score the better the sleep quality and the higher the score the worse the sleep quality. The opposite is true for arousal, lubrication, and orgasm. In this sense, for example, the correlation between sleep quality and orgasm (r = -0.310 p = 0.012) will indicate that the lower the score for sleep quality (better sleep) the higher the score for orgasm (better orgasm) (Table 7).

**Table 7:** Correlation matrix questionaries: Stress, anxiety, bruxism, TMD, sleep quality and IFSF questionnaire and their domains.

		Stress (PSS-14)	Anxiety (STAI)	Bruxism (CBA)	TMD (Fonseca Index)	Sleep Quality (Pittisburg)	Desire	Arousal	Lubrication	Orgasm	Satisfaction	Pain	FSFI
**Stress(PSS-14)**	Pearson Correlation	1	**,713** **	,235	,255	**,274** *	-,013	-,138	-,176	-,166	-,180	,079	,040
	P value		,000	,059	,040	,027	,917	,272	,160	,186	,159	,534	,781
**Anxiety(STAI)**	Pearson Correlation	**713** **	1	,217	,250*	,280*	-,108	-,180	-,176	-,170	-,170	-,077	-,122
	P value	,000		,083	,045	,024	,394	,151	,161	,175	,184	,543	,393
**Bruxism (CBA)**	Pearson Correlation	,**235**	,217	1	**,752** **	-,019	,016	,108	-,053	078	,003	,291*	,016
	P value	,059	,083		,000	,881	,898	,393	,674	,538	,979	,036	,911
**TMD (Fonseca Index)**	Pearson Correlation	,255	**,250** *	**,752** **	1	,250*	-,096	-,140	**-,254** *	-,114	-,171	,107	,039
	P value	,040	,044	,000		,044	,449	,266	,041	,365	,180	,395	,783
**Sleep quality (Pittisburg)**	Pearson Correlation	**,274** *	,280*	**-,**019	**,250** *	1	-,119	**-,335** **	**-,348** **	**-,310** *	-,212	-0,52	,056
	P value	**,027**	,024	**,881**	,044		,344	,006	,004	,012	,095	,680	,698
**Desire**	Pearson Correlation	-,013	-,108	,016	-,096	-,119	1	**,632** **	**,499** **	,614**	,552**	,069	,788**
	P value	,917	,394	,898	,449	,344		,000	,000	,000	,000	,585	,000
**Arousal**	Pearson Correlation	-,138	-,180	,108	-,140	**-,335** **	**,632** **	1	,888**	**,884** **	**,821** **	,389**	**,748** **
	P value	,272	,151	,393	,266	,006	,000		,000	,000	,000	,001	,000
**Lubrication**	Pearson Correlation	-,176	-,176	-,053	-,254*	**-,348** **	**,499** **	**,888** **	1	**,886** **	,793**	,370**	**,585** **
	P value	,160	,161	,674	,041	,004	,000	,000		,000	,000	,000	,000
**Orgasm**	Pearson Correlation	-,166	-,170	,078	-,114	-,310*	,614**	**,884** **	**,886** **	1	,839**	,313*	,813**
	P value	,186	,175	,538	,365	,012	,000	,000	,000		,000	,011	,000
**Satisfaction**	Pearson Correlation	-,180	-,170	,003	-,171	-,212	,552**	**,821** **	**,793** **	,839**	1	**,454** **	,808**
	P value	,159	,184	,979	,180	,095	,000	,000	,000	,000		,000	,000
**Pain**	Pearson Correlation	,079	,077	**,291** *	,107	-0,52	,069	**,389** **	,370**	,313**	**,454** **	1	-,141
	P value	,534	,543	,036	,395	,680	,585	,001	,000	,000	,000		,324
**FSFI**	Pearson Correlation	,040	-,122	,016	,039	,056	**,788** **	**,748** **	,585**	,813**	,808**	-,141	1
	P value	,781	,393	,911	,783	,698	,000	,000	,000	,000	,000	,324	

Correlation matrix between PPS-14 (Perceived Stress Scale), STAI (State-Trait Anxiety Index), Bruxism, CBA questionarie, Fonseca’s Anamnesis Index (TMD: temporomandibular disorders), Pittishburg Sleep Quality Index and FSFI (Female Sexual Function Index) and its domains: desire, arousal, lubrication, orgasm, satisfaction and pain.

Pearson Correlation

* p < .05

** p < .01

On the other hand, TMDs are correlated with lubrication r = -0.254 (p = 0.041). Bruxism is related to pain in sexual intercourse r = 0.261 (p = 0.036).

The sexual dysfunction index presents statistically significant relationships with desire r = 0.788 (p = 0.000), arousal r = 0.748 (p = 0.000), lubrication r = 0.585 (p = 0.000), orgasm r = 0.813 (p = 0.000) and sexual satisfaction r = 0.808 (p = 0.000). However, there is no statistically significant relationship with pain (Table 7).

## 4. Discussion

The results support the hypothesis since statistically significant relationships were found with respect to the muscle parameters measured and the questionnaires given to the subjects independently and related to each other.

While it is known that stress can produce changes in the human body (2) we must therefore consider the concept of myofascial chains, which explains that, the tension presented by one contractile district can have repercussions and influence on the other districts near and far away. In this case, the orofacial and pelvic floor musculature are distant, but they might be linked [39].

Regarding the correlation between the muscular parameters obtained with myotonometry, the relationship between the right temporalis and CFPF in terms of right temporalis relaxation and CFPF stiffness is noteworthy. As well as between both relaxation times. And the creep of the right temporal with the stiffness and elasticity of the CFPF. No previous studies have been found that address these relationships, and more studies with larger samples are needed to investigate this issue.

It should be noted that, just as there are previous studies assessing the CFPF [27] and masseter [17] with MyotonPRO independently, there are no studies assessing the temporalis muscle at present.

Like Herawati Kusuma et al, a statistically significant relationship between stress and sleep quality appears in this study. The study by Herawati Kusuma et al, assesses stress level with the scale (PSS-14) and sleep quality with the questionnaire (PSQI), the same questionnaires as in the present study [40]. In contrast to the systematic review of Luiz de Barreto Aranha Ricardo et al [41]. The present literature suggests that there is a significant association between stress and TMDs, which is considered a matter of controversy since the current evidence does not allow us to conclude the relationship [42].

Different studies, like the present one, propose the relationship between TMDs, anxiety, stress, sleep quality and bruxism [41, 42]. However, up to 2023, the literature is contradictory on this point since several reviews point out that the current level of evidence regarding the relationship between bruxism and TMDs is low [43, 44].

The search for a standard tool for the diagnosis of bruxism is being studied today but so far there is a consensus that the diagnosis of awake bruxism is based on self-reported diagnosis and electromyography which, according to SENIAM, are objective methods of diagnosis [26, 45]. Some authors mention that there is a high level of correlation between electromyography and MyotonPRO measurements [46].

In the present study, the subjective probability of suffering from bruxism was studied using the Self-Reported Bruxism Questionnaire [29] despite having a sample of healthy subjects with no previous diagnosis of bruxism. The analysis indicates that there is a value very close to p value 0.05 between bruxism and stiffness of the CFPF, therefore new studies with surface electromyography in the masseter are proposed to detect the presence of bruxism and subsequently relate it to the CFPF.

Regarding the Index of Sexual Dysfunction (IFSD), out of n = 65 study subjects, 17 women had not had sexual relations during the last month; therefore, to study sexual dysfunction, the final sample was n = 48. The IFSD and its domains (desire, arousal, lubrication, orgasm, satisfaction, and pain) do not present according to the analysis significant relationships with respect to the CFPF. This may be due to the fact that the chosen population is a healthy population, without PF dysfunctions; however, evidence indicates that there is a relationship between sexual dysfunction and PF dysfunctions [47].

Sleep quality is significantly related to excitation, lubrication, and orgasm, with better sleep quality being considered to affect greater excitation, lubrication, and better orgasm. Seehuus Martin et al, conducted a study with a sample n = 703, using the same PSQI and IFSF questionnaires, found a connection between poor sleep quality and sexual dysfunction [48]. Likewise, Kling Juliana M et al., indicate that poor sleep quality is related to sexual dysfunction in women, also using the PSQI and IFSF questionnaires, however, the population studied were women in menopausal stage [49].

TMDs correlated significantly and inversely with lubrication, indicating that the possible presence of TMDs is associated with worse lubrication. As bruxism seems to be related to pain in sexual intercourse, this relationship is positive, so the higher the level of possible presence of bruxism, the greater the pain in sexual intercourse. The latter correlations indicate a possible future line of research, although the possible cause is unknown, the results point to a possible relationship.

Just as bruxism seems to be related to pain in sexual relations, this relationship is positive, so the higher the level of possible presence of bruxism, the greater the pain in sexual intercourse. The latter correlations indicate a possible future line of research, although the possible cause is unknown, the results point to a possible relationship.

IFSF is significantly and positively related to desire, excitation, orgasm, and satisfaction. It should be considered that the higher the IFSF score, the better the sexual function, and therefore the higher the grade in the aforementioned domains [50]. However, no relationship has been found between the IFSF and pain during sexual intercourse. This may be due to the fact that pain is defined as a subjective experience, also, the sexual domain encompasses more aspects than penetration, it is possible that there are women whose sexual life is not focused on penetration and therefore sexual dysfunction is not related. Similarly, pain seems to be unrelated to desire. Sexual desire is stimulated by hormones and is considered innate [51]. However, an experience of pain may influence sexual desire by avoiding the situation; this can be explained by the fear-avoidance model of pain [52].

To the author’s knowledge, this is so far the only study that investigates the relationship between the orofacial region and the PF. Therefore, it is necessary to continue in this line of research and to broaden the knowledge of this area.

## 5. Limitations of the study

Among the limitations of the study, we can consider that the MyotonPRO measuring instrument has previous studies that measure masseter (23) and NFCP (25) muscle tone, but not in the temporalis muscle.

In addition, the sexual satisfaction questionnaire measures the different variables according to the last month, a condition that may affect subjects who have not had sexual relations during that period of time, which is equivalent to a score equal to 0, being excluded from the questionnaire (30).

It must be considered that the levels of stress and anxiety in the current population are extremely high, so individuals tend to adapt to very high levels (17), which may alter the final score of both questionnaires.

In addition, all the measurements performed evaluate the basal tone, not in contraction or relaxation.

## 6. Conclusion

In conclusion, the results of this study support the relationship between the orofacial region and the pelvic region. Furthermore, it is worth highlighting the association found between TMDs and vaginal lubrication, as well as bruxism and pain.

On the one hand, stress, anxiety, and poor sleep quality seem to be possible triggers of TMDs. On the other hand, good sleep quality seems to be associated with better arousal, lubrication and orgasm.

Further research and knowledge in this area should be considered due to the lack of previous evidence. New studies are proposed with the use of electromyography, as well as in subjects with previously diagnosed temporomandibular dysfunctions and in different population groups.

## Supporting information

S1 Data Original database. (XLSX)
